# Assessment of Point-of-Care Ultrasound (POCUS) Knowledge Among Lebanese Medical Residents

**DOI:** 10.7759/cureus.69205

**Published:** 2024-09-11

**Authors:** Yara A Mouawad, Fadi El Ters, Christeen Mina, Khalil Richa, Pascale Salameh, Ramzi Nakhle

**Affiliations:** 1 Emergency Department, Lebanese American University Medical Center, Beirut, LBN; 2 Primary Care and Population Health, University of Nicosia Medical School, Nicosia, CYP; 3 Public Health, Institut National de Sante Publique, Epidemiologie Clinique et Toxicologie (INSPECT-LB), Beirut, LBN; 4 Faculty of Pharmacy, Lebanese University, Beirut, LBN; 5 School of Medicine, Lebanese American University, Beirut, LBN

**Keywords:** lebanon, simulation training, medical education, emergency department, emergency medicine, point-of-care ultrasound (pocus)

## Abstract

Over the past few years, point-of-care ultrasound (POCUS) has emerged as a critical diagnostic tool in emergency medicine, providing real-time imaging at the bedside. This study aims to assess POCUS knowledge and competency among medical residents in Lebanon, identify possible gaps and deficiencies in their training, and recommend guidelines for further improvement of the curriculum in Lebanese medical schools and residency programs.

Our study reveals that 58.3% (N=119) of resident doctors from multiple specialties in Lebanon have only basic knowledge about POCUS, 19.6% (N=40) have no knowledge, and only 21.6% (N=44) have sufficient knowledge to perform diagnostic studies on a routine basis.

Lebanese medical residents currently possess suboptimal POCUS knowledge and proficiency due to disparities in training and educational obstacles. To address this, residency programs should focus on standardized POCUS training, simulation-based learning, and faculty development. This approach will help ensure residents gain the necessary skills to use POCUS effectively in clinical practice.

## Introduction

By offering quick and non-invasive diagnostic imaging, point-of-care ultrasound (POCUS) is quickly revolutionizing clinical practice in the acute care setting. It is becoming an increasingly used tool in clinical practice internationally. It is useful in many different areas of medicine, especially in critical care and emergency medicine [1]. As an extension of the physical exam, POCUS is a real-time bedside diagnostic tool that guides the diagnosis and treatment of medical conditions [2]. Moreover, it is cost-effective, has no radiation exposure, and improves the quality of care provided [3-5].

POCUS can rapidly diagnose life-threatening conditions such as aortic aneurysms and dissections, pericardial or pleural effusions, pneumothorax, and deep vein thrombosis among other diagnoses and emergencies. It can also be used to monitor clinical conditions, for instance, by evaluating left ventricular cardiac function and fluid status [3-6]. In addition, POCUS guides and improves the safety of many procedures such as regional anesthesia, injections into joints and soft tissues, central line placements, pericardiocentesis, and thoracentesis [4-6].

In many countries, the need to integrate POCUS training into the medical curricula has been recognized [1]. The knowledge level among medical professionals, particularly residents, in utilizing POCUS can vary significantly, depending on their specialties, the institution in which they work, and the training they have acquired. Without proper training, inappropriate POCUS use will lead to inaccurate diagnoses and consequently, patient harm [5,7]. In fact, ultrasound education and training have started to be incorporated into some undergraduate medical education curricula as well as residency programs [4-6].

A 2021 study done in Madrid showed that POCUS is frequently new to first-year residents, likely due to a lack of training during their medical education. However, when they gain experience in POCUS-equipped emergency rooms, they realize the importance of POCUS and show interest in acquiring proficiency in its use [8].

In Lebanon, there is limited evidence regarding how POCUS is taught in medical schools or integrated into residency training programs. To design effective educational interventions, it is essential to assess the current POCUS knowledge and competency levels of medical residents across all specialties. Understanding these factors is crucial for optimizing POCUS integration into routine clinical practice and improving patient outcomes. This study specifically aims to identify potential gaps and areas for improvement in medical training within Lebanon and is intended solely for residents of all medical specialties.

## Materials and methods

Study design and participants

The study was conducted using a cross-sectional design to determine the knowledge of Lebanese medical residents on POCUS. Data collection was conducted from January to May 2024. Included in the study were 204 resident doctors from all post-graduate training years, across major medical schools in Lebanon: Lebanese American University (LAU), Lebanese University (LU), American University of Beirut (AUB), Université Saint Joseph (USJ), Beirut Arab University (BAU), University of Balamand (UOB), Université Saint Esprit de Kaslik (USEK).

Data collection and exclusion criteria

A Google Form survey (https://acrobat.adobe.com/id/urn:aaid:sc:ap:c362f430-4e17-4ca8-94ae-4a02282ba27c?viewer%21megaVerb=group-discover), written in English, was distributed via WhatsApp to all current residents in the major Lebanese medical schools previously mentioned. International medical graduates currently rotating with Lebanese medical graduates in the same residency programs were excluded from the study.

Participants were asked to complete the questionnaire anonymously. Participation was completely voluntary, and data collection was entirely anonymous. After being instructed about the nature and purpose of the survey, all respondents provided informed consent and were given the option to withdraw at any time. The questionnaire's development was guided by the study's objectives and informed by a thorough literature review.

The first part of the questionnaire focused on participants’ demographic information, including their specialty, level of training, and the university from which they graduated. In the second part, participants were asked to assess their knowledge and training in POCUS; they were asked about its integration into their medical education and residency training, the frequency of its use in their medical training, and their accessibility to an Ultrasound machine. After that, participants were asked to rate their skills in using POCUS, including how comfortable they feel performing the exam and their proficiency in conducting diagnostic studies. Finally, the questionnaire explored participants’ opinions on the impact of POCUS on their practice and medical specialty, and their interest in improving their POCUS skills with further workshops/training.

Data analysis

The collected data were analyzed using SPSS software, version 28.0. For descriptive analysis, frequency and percentage are used for categorical variables, and median and IQR for quantitative variables. For the bivariate analysis of categorical variables, the Chi-squared test was used, and the Fisher exact test in cases of expected values less than 5. In all cases, a p-value lower than 0.05 was considered significant.

Ethical considerations

Both the survey and study conduction were approved by the Institutional Review Board (IRB) of the LAU. Date of approval: 08/02/2024, code: 2024-IRB-04. A consent form is included at the beginning of the questionnaire. All the data collected during the research were anonymous and participants could not be identified. Additionally, only authorized research team members had access to the data.

## Results

Demographic data

The survey was sent to a total of three hundred and eighty residents, of which two hundred and four responded, resulting in a response rate of 53.68%. Among the respondents, there were 70 first-year residents, 58 second-year residents, 40 third-year residents, 15 fourth-year residents, 2 fifth-year residents, and 19 medical fellows (Table 1).

**Table 1:** Percentage distribution of medical residents by year. PGY: Post-graduate year.

Medical Doctors	Frequency	Percent
Medical Fellow	19	9.3
Medical Resident: PGY1	70	34.3
Medical Resident: PGY2	58	28.4
Medical Resident: PGY3	40	19.6
Medical Resident: PGY4	15	7.4
Medical Resident: PGY5	2	1.0
Total	204	100.0

The median age was 27 (range 23-33). Fifty percent of the sample were females (N=102). Seventy-six percent were from medical specialties (N=155), and twenty-four percent were from surgical specialties (N=49).

Knowledge levels about POCUS

Our study reveals that 58.3% (N=119) of resident doctors in Lebanon have basic knowledge when using POCUS but need supervision, 19.6% (N=40) have no knowledge, and only 21.6% (N=44) have sufficient knowledge to perform diagnostic studies on a routine basis. 97.2% (N=68) of first-year residents and almost 61% (N=82) of senior residents had no or very low knowledge about POCUS (p-value < 0.001).

**Figure 1:**
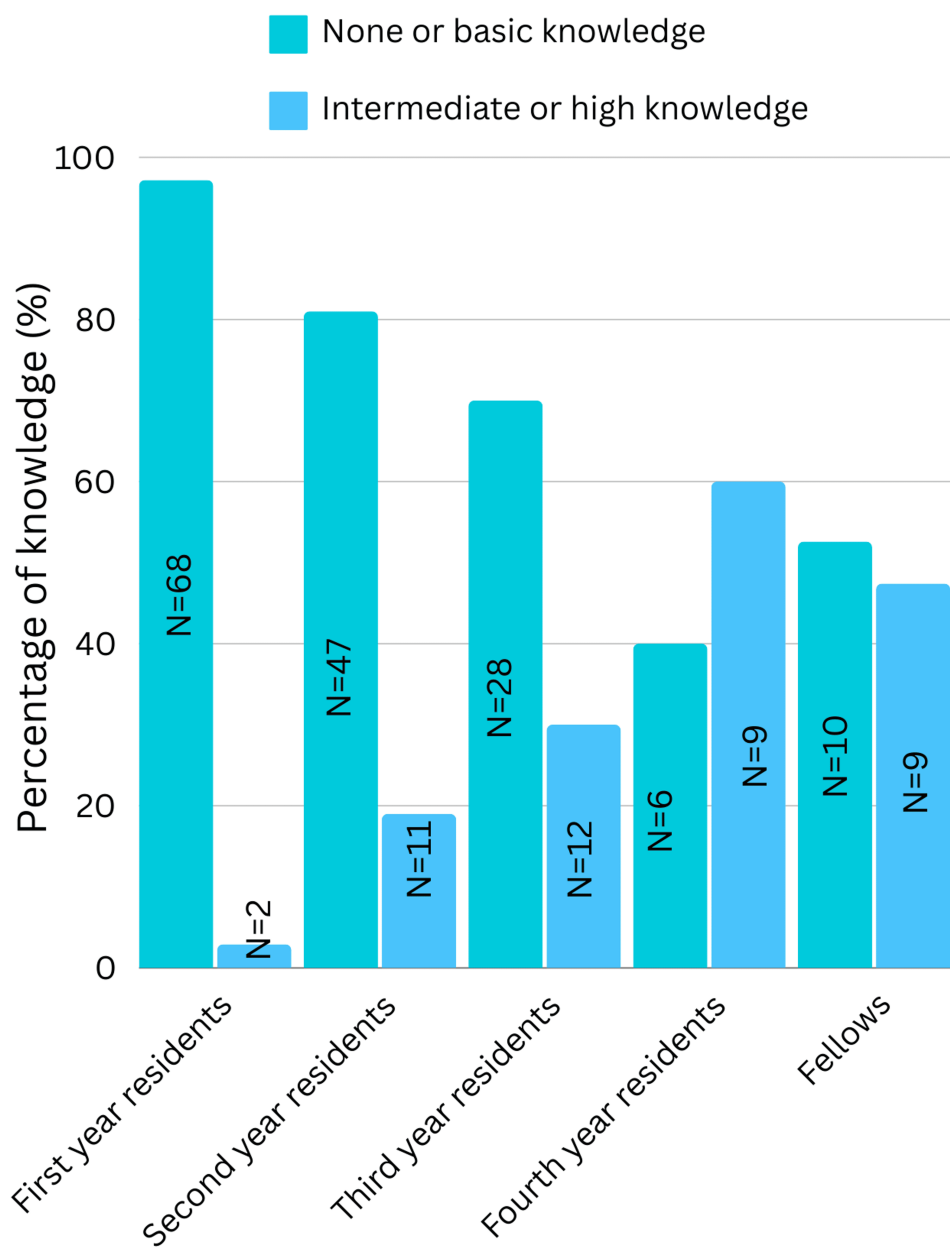
Knowledge levels of POCUS among medical residents and fellows. POCUS: Point-of-Care Ultrasound.

As shown in Figure 1, residents with higher levels of training demonstrate greater familiarity with POCUS.

Within the medical specialties, 77.4% (N=120) had no or basic knowledge about POCUS (p-value 0.886) compared to 79.6% (N=39) in surgical specialties (p-value = 0.865).

Training and curriculum integration

A total of 93.1% (N=190) of resident doctors emphasized how valuable POCUS is for their specialty now or in the future, highlighting why it's crucial to incorporate POCUS training during medical school and residency.

A total of 80.4% (N=164) of resident doctors stated that they didn't receive POCUS education during their medical school. 62.7% (N=128) haven’t received POCUS training during their residency.

Among residents who received POCUS training, 44.6% (N=25) feel confident enough to routinely perform diagnostic studies. In comparison, only 12.8% (N=19) of those without POCUS training share the same level of confidence (p-value <0.001).

There was a difference in POCUS training provided between universities, but overall, the levels of training were low. For instance, at the Lebanese American University (LAU), only 4.2% (N=2) of students reported that a specific course was given focusing on the use of POCUS machinery, its application, and benefits. Similarly, at the Lebanese University (LU), only 4% (N=3) of students reported the same (p-value = 0.133).

Interest in POCUS workshops and courses

Among resident doctors, 85.3% (N=174) are keenly interested in attending POCUS workshops, highlighting the importance of incorporating POCUS education into medical school courses and residency workshops.

**Figure 2:**
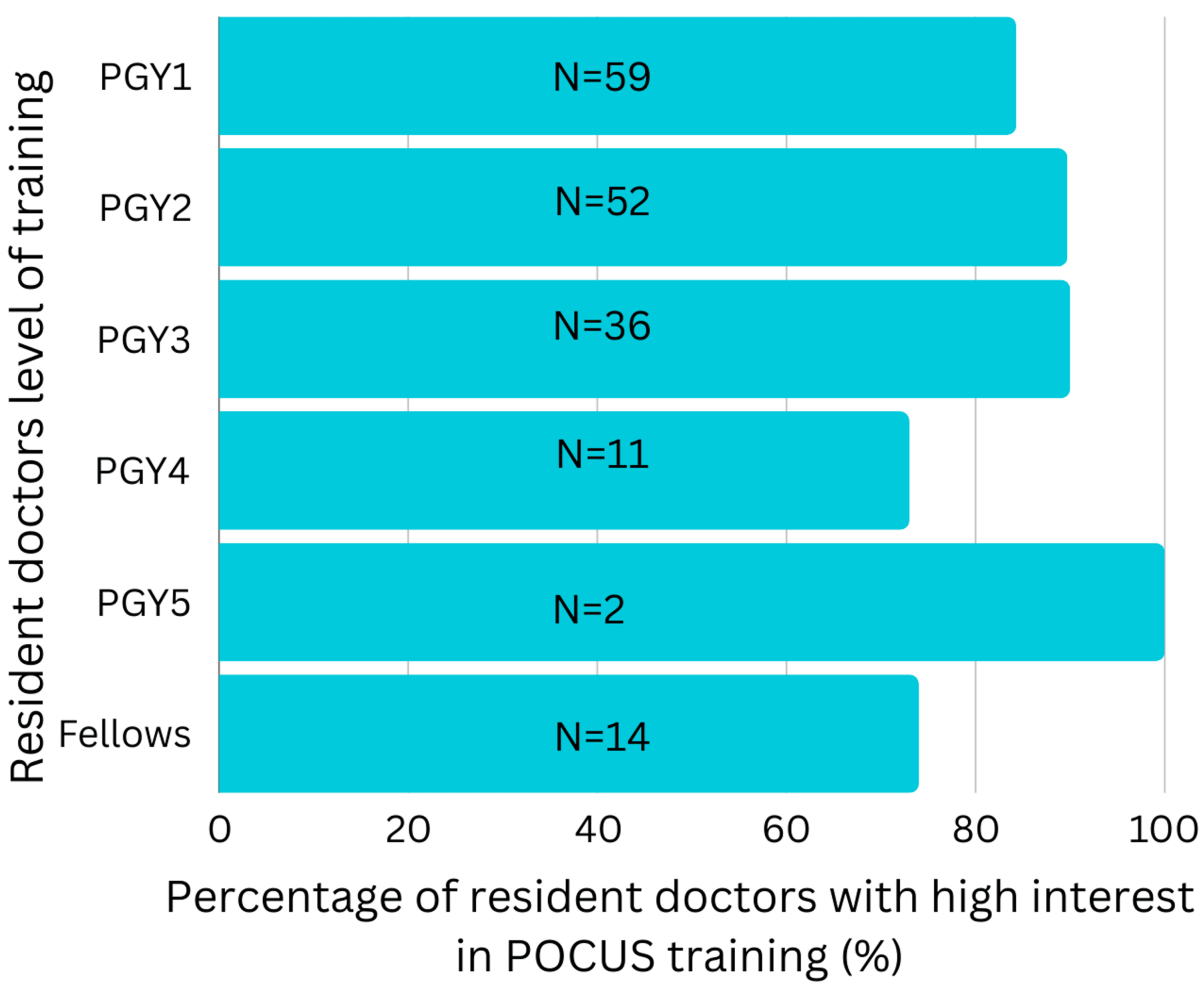
Percentage of resident doctors with high interest in POCUS training. POCUS: Point-of-Care Ultrasound; PGY: Post-graduate year.

To note, resident doctors show high interest in attending POCUS workshops irrespective of their level of training, as indicated in Figure 2 (p-value = 0.424).

Access to ultrasound machine

Our study showed that 79.9% (N=163) of resident doctors had access to an ultrasound machine in their hospitals, whereas only 39.7% (N=81) used it in their clinical practice.

## Discussion

POCUS knowledge and training

POCUS knowledge and training remain limited among Lebanese medical residents, with only 21.6% (N=44) feeling confident in performing basic POCUS evaluations independently. Concerningly, 61% (N=82) of senior residents reported knowing little or nothing about POCUS. Among those who received POCUS training, 44.6% (N=25) felt confident enough to routinely perform diagnostic studies, in contrast to just 12.8% (N=19) of those without training.

These findings highlight a lack of confidence and knowledge in POCUS among residents in Lebanon, largely due to insufficient training during medical school and early residency. According to El Majzoub IA et al., only 40% of Lebanese emergency physicians had received official POCUS training. A significant obstacle to POCUS adoption in Lebanon was the absence of formal training [9]. Williams JP et al., through a cross-sectional study involving over 100 chiefs across various specialties, identified inadequate training and the lack of protected time for training as the most frequently mentioned barriers to POCUS utilization [10]. Interestingly, no significant difference was found when comparing POCUS knowledge between medical and surgical specialties (p-values 0.886 and 0.865, respectively). Additionally, our study reveals differences in POCUS training between universities in Lebanon. Some programs provide minimal or no formal training, while others offer more structured POCUS curricula. The proficiency levels of residents who received structured POCUS training were greater than those of their counterparts who did not receive formal training.

Barriers to effective POCUS training

POCUS training is hindered by several factors including residency program time constraints, a shortage of trained instructors, and restricted access to ultrasound machines. This is further complicated by the absence of standardized curricula and assessment tools. Other barriers include senior faculty members' resistance to change and lack of funding for important POCUS tools and equipment [11,12]. Institutional dedication and funding are needed to remove these obstacles. Our study showed that 79.9% (N=163) of resident doctors had access to an ultrasound machine in their hospitals, whereas only 39.7% (N=81) used it in their clinical practice. This is mostly due to the lack of training and confidence in performing POCUS evaluations.

Importance of standardized training

Based on our findings, 93.1% (N=190) of resident doctors emphasized the importance of POCUS in their practice, and a high prevalence of interest in POCUS workshops (85.3%, N=174) was observed among resident doctors across all training levels, emphasizing a strong demand for POCUS education within medical training programs. These results are consistent with those of Oh J et al. and Thomas VK, who have highlighted the importance of POCUS in improving patient care and diagnostic accuracy in emergency situations [13,14].

Consequently, standardized POCUS training programs should be implemented in all Lebanese residency programs to narrow the knowledge gap. These must consist of didactic sessions, hands-on training, and ongoing competency evaluations. Prioritizing POCUS integration within residency curricula will help ensure that every resident reaches a minimum required basic level of proficiency [15]. According to Martin R et al., standardized POCUS education should be incorporated into both undergraduate and graduate medical education programs, as it is crucial to the medical training curriculum [15]. Even brief, standardized instruction, such as a one-hour session for third-year medical students during a surgical clerkship, has been shown to significantly improve competency in performing and interpreting ultrasound images [16].

Role of simulation-based learning

The use of simulation-based learning has shown promise for enhancing residents' POCUS skills. Research by Hagood NL et al. shows that students' confidence and diagnostic accuracy can be greatly enhanced by simulation-based training. Incorporating training sessions into the curriculum will provide students with a safe and secure environment in which they can practice and refine their skills [17]. According to research by Nti B et al., POCUS skill retention was stronger when simulation-based training was paired with traditional hands-on instruction. Simulation-trained residents performed better on practical exams and in real-life clinical settings [18].

Faculty development and mentorship

A key component of the success of POCUS training programs is faculty development. Residency programs should invest in training faculty members to become proficient POCUS instructors [11,12]. Furthermore, implementing a mentorship program in which experienced faculty members support residents can enhance learning outcomes and promote a culture of continuous improvement. Russell FM et al. found that having dedicated ultrasound faculty significantly improves patient outcomes and the quality of POCUS education [19]. POCUS-versed faculty members can guarantee that residents receive adequate training through consistent training and mentorship [11].

Limitations

The study has several limitations that should be acknowledged. First, there is a potential for social desirability bias, as residents might misinterpret or overstate their knowledge or training in POCUS. Additionally, the cross-sectional design of the study offers only a snapshot of POCUS knowledge at a specific point in time, which may not reflect changes that could occur with further training or experience. Another limitation is the generalizability of the findings, as the study is focused on residents in specific hospitals in Lebanon, and the results may not be applicable to other hospitals or healthcare systems. Lastly, there is the possibility of instrument bias, as the questionnaire used was developed by the researchers and has not been validated, which may affect its accuracy in measuring the intended variables.

## Conclusions

Due to disparities in training and educational obstacles, Lebanese medical residents currently have suboptimal POCUS knowledge and proficiency.

Standardized POCUS training, simulation-based learning, and faculty development are the three main areas on which Lebanese medical residency programs need to focus to overcome this knowledge gap. By doing this, we will ensure that residents have the fundamental expertise and skills required to apply POCUS appropriately in their clinical practice.
